# Completeness of spontaneously reported adverse drug reactions in 4 databases

**DOI:** 10.1002/bcp.70182

**Published:** 2025-07-28

**Authors:** Mohammed Gebre Dedefo, Gizat M. Kassie, Eyob Alemayehu Gebreyohannes, Renly Lim, Elizabeth Roughead, Lisa Kalisch Ellett

**Affiliations:** ^1^ Quality Use of Medicines and Pharmacy Research Centre UniSA Clinical and Health Sciences, University of South Australia Adelaide SA Australia; ^2^ South Australian Health and Medical Research Institute Adelaide SA Australia; ^3^ College of Medicine and Public Health, Flinders University Adelaide SA Australia; ^4^ Centre for Optimisation of Medicines School of Allied Health, The University of Western Australia Perth WA Australia

**Keywords:** adverse drug reaction reporting systems, drug safety, drug‐related side effects and adverse reactions, pharmacovigilance, postmarketing surveillance

## Abstract

**Aims:**

To assess the completeness of information provided in adverse drug reaction (ADR) reports in 4 spontaneous report databases.

**Methods:**

The study was conducted using freely accessible ADR reports from the Canada Vigilance Adverse Reaction Online Database, the US Food and Drug Administration Adverse Event Reporting System (FAERS) database, the UK Yellow Card Scheme and the Danish Medicines Agency ADR Database, covering the period 2014–2023. The variables used to evaluate the completeness of ADR reports were selected based on the vigiGrade completeness tool. Descriptive statistics were used to report the completeness of information in each ADR report, while chi‐square tests were used to analyse differences in completeness by seriousness of report.

**Results:**

In total, 290 079 individual case safety reports were analysed: 4289 from the Canadian database; 269 763 from the FAERS database; 13 624 from the UK Yellow Card Scheme; and 2403 from the Danish database. The most frequently completed information was the primary reporter, with nearly 100% completeness. The most frequently omitted information was the route of administration, with completion scores of 44% in the UK Yellow Card Scheme, 55% in the Danish database and 58% in the Canadian database. In FAERS, the event date was the most frequently omitted information with a completeness rate of 50%.

**Conclusion:**

The completeness of information varied across different variables, with information frequently omitted for route of administration and event date. Enhancing awareness about providing complete information during ADR reporting is essential for collecting complete data and allowing signal detection.

**What is already known about this subject**

- The completeness of each spontaneous adverse drug reaction (ADR) report is an important factor in conducting a causality assessment between the suspected medicine and the reported ADR. However, incomplete spontaneous ADR reports present a challenge in undertaking the causality assessments.
- Previous studies have highlighted variations in the completeness of information reported to regulators.

**What this study adds**

- Information with the highest completeness of reports in the ADR reporting databases included primary reporter, outcome and sex.
- Information frequently omitted in the ADR reports included route of administration, event date, dose and indications.
- This study shows that between 30 and 51% of ADR reports included in the analysis had information recorded in all of the available variables within the databases used for undertaking causality assessments between the medicine and the reported ADR.

## 1 INTRODUCTION

Spontaneous adverse drug reaction (ADR) reporting constitutes a system wherein case reports of suspected ADRs are submitted by healthcare professionals, pharmaceutical manufacturers or consumers to pharmacovigilance centres.^1^ It serves as the mainstay for national and international medicine safety evaluation in the postapproval phase.^1^, ^2^ It is a vital part of medicine safety and pharmacovigilance,^3^ with the analysis of reports generating signals of previously unknown ADRs^4^, ^5^ for further investigation. Using the reported ADR information, causality assessment methods can be employed to determine whether there is a signal between the medicine and the reported ADR, including the Naranjo algorithm,^6^ the World Health Organization (WHO)–Uppsala Monitoring Centre scale,^7^ and probabilistic/Bayesian methods.^8^, ^9^ The quality of the information included in ADR reports plays a critical role in the process of adverse event signal detection and the completeness of each report is an important factor when undertaking causality assessment of reported reactions with suspected medicines.^10^

Completeness of ADR reports is a quality indicator that refers to the extent to which the report contains all of the necessary and relevant information about the ADR.^11^ The WHO recommends that ADR reports should collect information on the patient (sex, age, medical history), adverse event (severity, start date, treatment and outcome), suspected medicine (name, dose, route of administration, start and stop dates, and indication for use), concomitant medications, risk factors and reporter type (consumer or healthcare professional).^10^

There are several tools to assess the completeness of spontaneous ADR reports. One of these is the vigiGrade Completeness score,^11^ a validated tool developed by WHO–Uppsala Monitoring Centre to assess the completeness of reports in VigiBase, the WHO global database of reported suspected ADRs.^12^ The vigiGrade completeness tool includes the variables time‐to‐onset, indication, outcome, sex, age, dose, country, primary reporter, report type and comments.^11^ Another validated assessment tool is the clinical documentation tool (ClinDoc) developed by a panel of pharmacovigilance experts to assess the level of clinical information reported in ADR reports.^13^ ClinDoc tool includes a description of the ADR, chronology of the ADR, suspected medicine and patient characteristics to evaluate the completeness of ADR reports.^13^ Other studies have used the good pharmacovigilance practices criteria assessment tool, which defines mandatory variables for completion in ADR reports, including patient information (initials, date of birth, sex), suspected ADR(s), event date, suspected medicine(s) and start date; and nonmandatory, variables including the patient's medical history, concomitant medicine(s), clinical course or ADR outcome, and documentation of the indication diagnosis.^14^, ^15^, ^16^

The completeness of ADR reporting in previous studies conducted in Brazil, France, South Africa, Japan, Portugal and Spain varied from 4 to 55% depending on the study and database analysed.^14^, ^15^, ^17^, ^18^, ^19^, ^20^ The results of these studies indicate that the completeness of spontaneous ADR reports is often insufficient for subsequent causality assessment of the association between exposure to medicines and adverse events.^21^ Different countries use distinct reporting forms and guidelines,^22^ which may influence the completeness of ADR reporting. For this reason, it is important to investigate what information elements are well documented and what information is frequently omitted in ADR reporting databases of different countries.

This study aimed to assess the level of completeness of information of ADR reports in 4 different databases. We limited the study to the glucose‐lowering medicines: sodium–glucose cotransporter 2 inhibitors (SGLT‐2is), glucagon‐like peptide 1 receptor agonists (GLP‐1RA) and dipeptidyl peptidase‐4 inhibitors (DPP‐4i) as a case study example to answer our research question. We chose these medicines because they are widely used worldwide, and had similar regulatory approval timelines across different countries.^23^, ^24^, ^25^, ^26^

## 2 METHODS

### 2.1 Data sources and study design

A retrospective study was conducted using publicly accessible ADR reports from the Canada Vigilance Adverse Reaction Online Database,^27^ the Food and Drug Administration (FDA) Adverse Event Reporting System (FAERS) of the USA,^28^ the Yellow Card Scheme of the UK^29^ and the Danish Medicines Agency's Adverse Drug Reaction Database.^30^ We used the generic names of medicines from the class of SGLT‐2is, GLP‐1RA and DPP‐4i (Table S1) to identify ADR reports for these medicines in the databases. The study period was 1 January 2014, to 31 December 2023. We limited analysis to spontaneous reports; ADR reports derived from research studies and published literature were excluded.

### 2.2 Removal of duplicate reports

Duplicate reports were excluded from the analysis. In the Canada Vigilance ADR reporting database, duplicate reports are designated as duplicates in a specific variable, the Link/Duplicate report information field.^31^ In reports with this designation in the Canada Vigilance ADR reporting database, the most recent report was selected and included into the analysis, while other duplicates were excluded. Duplicate reports were identified in the FAERS database if they had the same case identifier, or if they had differing case identifiers but identical information reported for 9 other variables, including suspected product, reactions, sex, event date, patient age, reporter type, initial date received, indications and country. This method of duplicate identification in the FAERS data has been described previously.^32^, ^33^, ^34^ Removal of duplicate reports was not required for the UK Yellow Card Scheme data or the Denmark ADR reporting database, as duplicate reports are removed from these data sources by the regulatory authority staff before the reports are made publicly available.^35^, ^36^, ^37^, ^38^

### 2.3 Study variables and completeness assessment

We identified the variables used in the vigiGrade completeness score within the databases used for the study,^11^ along with availability of variables important for assessing causality, as outlined by the WHO.^10^ Additionally, we included the variable *seriousness* to assess whether completeness differed based on the seriousness of the report. The regulatory authority categorizes the ADR reports as either serious or nonserious based on the information provided by the reporter. An ADR is classified as serious if the reporter indicates that it involves death, a life‐threatening condition, inpatient hospitalization or prolonged hospitalization, significant disability or incapacity, a congenital anomaly or birth defect, or any other medically important event.^16^, ^28^, ^31^ A summary of the availability of each of the variables in the data sources is provided in Table 1. The vigiGrade completeness score ranges from 0.07 to 1, with scores > 0.8 indicating a well‐documented report.^11^ The vigiGrade completeness score applies penalties for missing information when assessing the completeness of ADR reports. Specifically, the missing of time‐to‐onset information results in a 50% penalty (score multiplied by a factor 0.5), while each missing variable among age, sex, outcome or indication incurs a 30% penalty (score multiplied by a factor 0.7). Furthermore, the omission of any other individual variable included in the vigiGrade completeness tool leads to a 10% penalty (score multiplied by a factor 0.9; Table S2).^11^ Our initial assessment highlighted that the vigiGrade completeness score was not suitable for use in the present study, due to the unavailability of key variables in many of the data sources. Time‐to‐onset, which carries the maximum penalty in the vigiGrade scoring system, is not available in the publicly accessible ADR reporting databases included in our study. Moreover, we included additional variables considered important by the WHO, such as event date and route of administration, which are not part of the vigiGrade variables. We therefore used an alternative approach for assessing the completeness of ADR reports, which has been described previously.^39^ For each variable listed in Table 1 that was available in the dataset for that country, we determined whether data were recorded or not. The percentage of reports in each dataset with each variable reported was calculated, and the overall completeness score for reports was also calculated for each dataset. The total number of variables recorded in a given dataset was used as the denominator for the overall completeness score calculation and the number of variables with data recorded was the numerator. Results were expressed as a percentage. For example, in the UK Yellow Card Scheme database, we considered 5 variables in the completeness assessment (Table 1) and if the report included data for 4 of those 5 variables an overall completeness score of 80% was calculated. The average overall completeness scores in percentage form for reports within each database was calculated. In addition, we considered the proportion of all ADR reports within each database that had information recorded for each of the available variables.

**TABLE 1:** List of variables included for assessing completeness of ADR reports in 4 databases.

Variables		Canada ADR reporting database	FAERS database	UK yellow card scheme	Danish ADR reporting database
**Variables of vigiGrade completeness tool** ^**#**^	Time‐to‐onset	X	X	X	X
	Indication	✔	✔	X	X
	Outcome	✔	✔	✔	✔
	Sex	✔	✔	✔	✔
	Age	✔	✔	✔	✔
	Dose	✔	X	X	X
	Country	X*	✔	X*	X*
	Primary reporter	✔	✔	✔	✔
	Comments	X	X	X	X
**Additional variables assessed for completeness**	Event date	X	✔	X	X
	Route of administration	✔	X	✔	✔
**Other variables included in the study**	Seriousness	✔	✔	✔	✔

Abbreviations: ADR, adverse drug reaction; FAERS, Food and Drug Administration Adverse Event Reporting System.

✔ The data are available for that country.

X Although the information is included in ADR reporting forms, it is not recorded in the publicly available database for that country.

X* These data sources only include domestic reports,^27^, ^29^, ^30^ making the variable *country* noninformative.

^#^ Report type was not included in the table under the variables of vigiGrade completeness tool because we have excluded it from the completeness assessment, given that the focus of this study is on spontaneous ADR reports.

### 2.4 Data analysis

Descriptive statistics were used to describe the distribution of reports by year, patient sex, patient age, primary reporter and seriousness of the report. Trends in the completeness of information in ADR reports from the 4 countries, covering the period from 2014 to 2023, were presented. As the variables included in each database varied, completeness was calculated for reports within each database. The completeness of ADR reports was stratified by seriousness of the report.

A chi‐square test was conducted to determine whether statistically significant differences existed between the completeness of reports for age, sex, outcome, indication, dose, route of administration, event date, country and whether the reports were classified as nonserious or serious. The analysis was performed using Statistical Package for Social Sciences (SPSS) version 29 (IBM Corporation, New York, NY, USA), with a *P*‐value of <.001 considered statistically significant.

## 3 RESULTS

**FIGURE 1:**
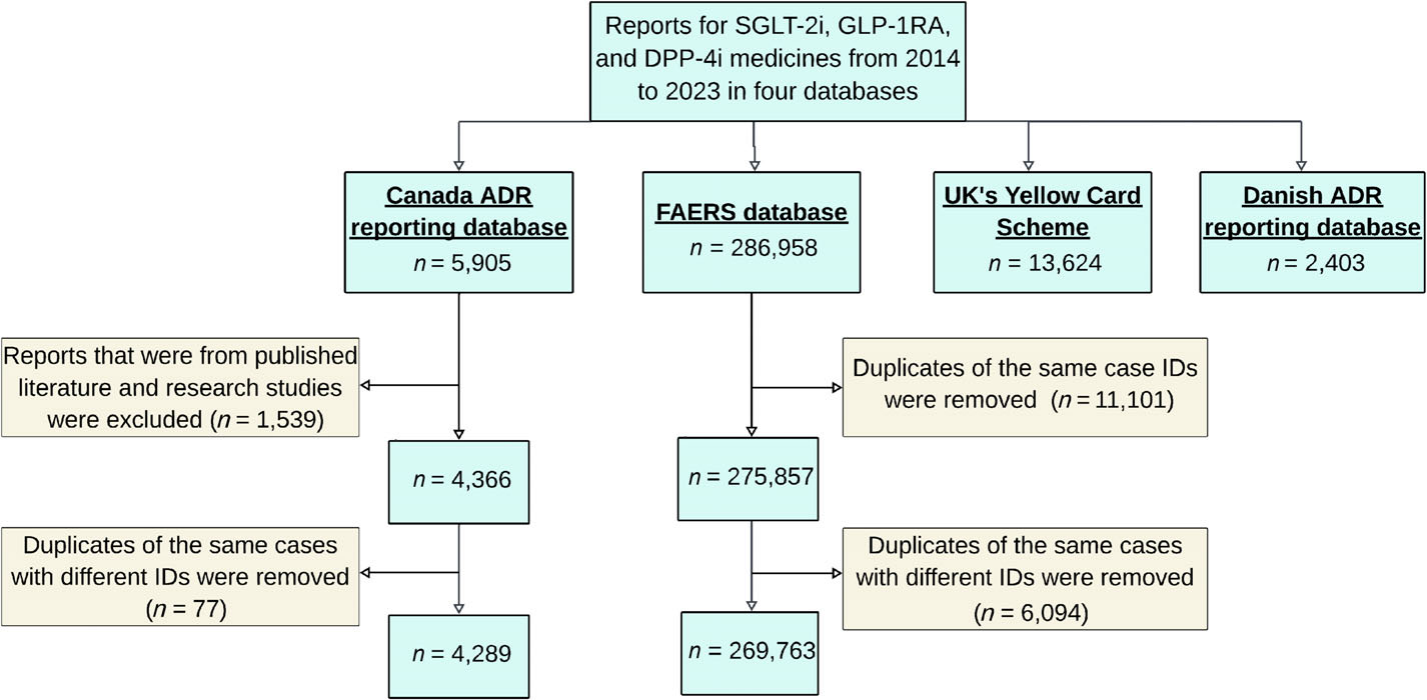
Flowchart of selection of the cases and exclusion criteria of adverse drug reaction (ADR) reports involving sodium–glucose cotransporter 2 inhibitor (SGLT‐2i), glucagon‐like peptide 1 receptor agonist (GLP‐1RA) and dipeptidyl peptidase‐4 inhibitor (DPP‐4i) medicines from 2014 to 2023 in 4 databases.

Totals of 4289 reports from the Canadian ADR reporting database, 269 763 reports from FAERS, 13 624 reports from the UK Yellow Card Scheme and 2403 reports from the Danish ADR reporting database met the inclusion criteria for the analysis (Figure 1).

### 3.1 Distribution of reports by year, sex, age, primary reporter and seriousness

The majority of primary reporters were healthcare professionals in Canada ADR reporting database (80%), the UK Yellow Card Scheme (85%) and Danish ADR reporting database (54%), whereas consumers constituted the majority of reporters in the FAERS database (58%). Approximately half of the ADR reports in the 4 databases were reported for female patients. Among the ADR reports with complete information for age, reports were predominantly made for patients aged ≥45 years, with more than 80% of reports from Canada ADR reporting database, the FAERS database and the UK Yellow Card Scheme and 70% of reports from Denmark made for patients aged ≥45 years. The majority of reports were classified as serious reports in the Canada ADR reporting database (83%) and the UK Yellow Card Scheme (57%), whereas nonserious reports comprised the majority of reports in the FAERS database (56%) and Danish ADR reporting database (74%; Table 2).

**TABLE 2:** Distribution of reports by year of the report, patient sex, patient age, primary reporter and seriousness of the report from 2014 to 2023 in the 4 databases.

Variables		Canada ADR reporting database (*N* = 4289)	FAERS database (*N* = 269 763)	UK yellow card scheme (*N* = 13 624)	Danish ADR reporting database (*N* = 2403)
Year of report	2014	126 (2.9%)	13 191 (4.9%)	610 (4.5%)	103 (4.3%)
	2015	309 (7.2%)	27 057 (10.0%)	889 (6.5%)	83 (3.5%)
	2016	356 (8.3%)	26 770 (9.9%)	1132 (8.3%)	88 (3.7%)
	2017	365 (8.5%)	23 868 (8.8%)	1131 (8.3%)	80 (3.3%)
	2018	402 (9.4%)	28 209 (10.5%)	1124 (8.3%)	93 (3.9%)
	2019	480 (11.2%)	28 701 (10.6%)	1212 (8.9%)	249 (10.4%)
	2020	485 (11.3%)	28 444 (10.5%)	904 (6.6%)	198 (8.2%)
	2021	489 (11.4%)	30 010 (11.1%)	1448 (10.6%)	292 (12.2%)
	2022	428 (10.0%)	30 194 (11.2%)	1917 (14.1%)	308 (12.8%)
	2023	849 (19.8%)	33 319 (12.4%)	3257 (23.9%)	909 (37.8%)
Patient sex	Male	1881 (43.9%)	112 609 (41.7%)	5852 (43.0%)	909 (37.8%)
	Female	2134 (49.8%)	134 840 (50.0%)	7189 (52.8%)	1356 (56.4%)
	Missing	274 (6.4%)	22 314 (8.3%)	583 (4.3%)	138 (5.7%)
Patient age (years)	<18	48 (1.1%)	379 (0.1%)	38 (0.3%)	5 (0.2%)
	18–44	391 (9.1%)	12 905 (4.8%)	2020 (14.8%)	551 (22.9%)
	45–54	530 (12.4%)	23 087 (8.6%)	2698 (19.8%)	486 (20.2%)
	55–64	889 (20.7%)	39 592 (14.7%)	2830 (20.8%)	409 (17.0%)
	65–74	860 (20.1%)	39 367 (14.6%)	1933 (14.2%)	299 (12.4%)
	75–84	466 (10.9%)	18 675 (6.9%)	548 (4.0%)	80 (3.3%)
	≥85	112 (2.6%)	4412 (1.6%)	50 (0.4%)	1 (0.0%)
	Missing	993 (23.2%)	131 346 (48.7%)	3507 (25.7%)	572 (23.8%)
Primary reporter	Consumer	863 (20.1%)	155 019 (57.5%)	1739 (12.8%)	884 (36.8%)
	Healthcare professional	3418 (79.7%)	114 104 (42.3%)	11 565 (84.9%)	1306 (54.3%)
	Both consumer and healthcare professional	–	–	320 (2.3%)	213 (8.9%)
	Missing	8 (0.2%)	640 (0.2%)	0 (0.0%)	0 (0.0%)
Seriousness	Nonserious	724 (16.9%)	150 120 (55.6%)	5798 (42.6%)	1768 (73.6%)
	Serious	3565 (83.1%)	119 643 (44.4%)	7826 (57.4%)	635 (26.4%)
Medicine suspected of causing the ADR*	SGLT‐2i	2122 (49.5%)	75 620 (28.0%)	6210 (45.6%)	285 (11.9%)
	GLP‐1RA	1474 (34.4%)	151 149 (56.0%)	5576 (40.9%)	2023 (84.2%)
	DPP‐4i	766 (17.9%)	32 470 (12.0%)	1838 (13.5%)	95 (3.9%)
	Fixed‐dose combination	0 (0.0%)	21 119 (7.8%)	0 (0.0%)	0 (0.0%)

Abbreviations: ADR, adverse drug reaction; DPP‐4i, dipeptidyl peptidase‐4 inhibitor; FAERS, Food and Drug Administration Adverse Event Reporting System; GLP‐1RA, glucagon‐like peptide 1 receptor agonist; SGLT‐2i, sodium‐glucose cotransporter 2 inhibitor.

– Variable not reported in that database.

* More than 1 suspected medicine is allowed in ADR reports in the Canadian and FAERS databases.

SGLT‐2is were most frequently suspected of causing the ADR in reports from the Canadian database (50%, *n =* 2122) and the UK Yellow Card Scheme (46%, *n =* 6210). GLP‐1RAs were the most frequently reported medicines suspected of causing the ADR in the FAERS database (56%, *n =* 151 149) and the Danish database (84%, *n =* 2023). DPP‐4is accounted for a smaller proportion of suspected medicines in reports across all databases, ranging from 4% in the Danish database to 18% in the Canadian database (Table 2).

### 3.2 Completeness of information of ADR reports

**FIGURE 2:**
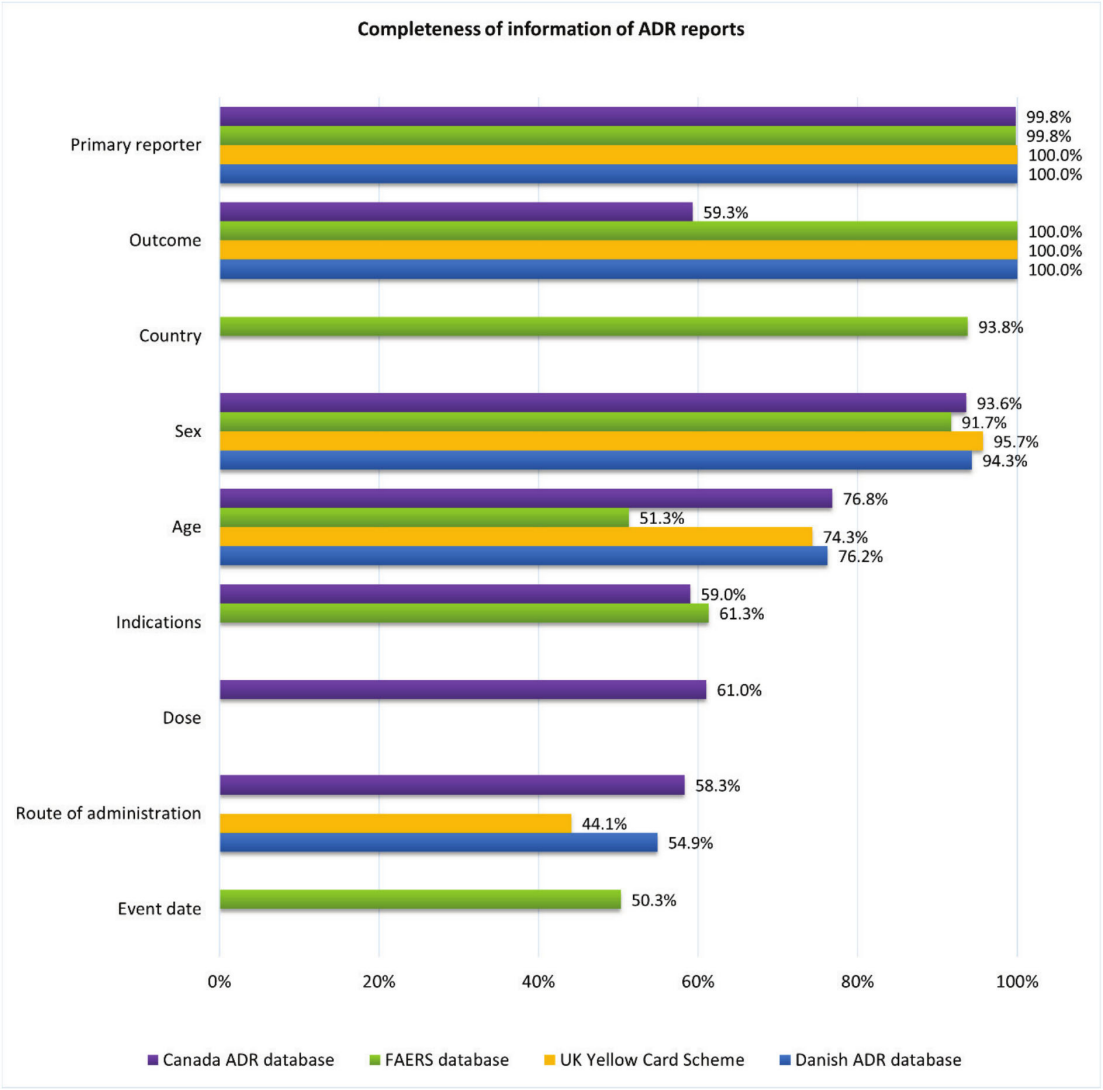
Completeness of adverse drug reaction (ADR) reports for sodium–glucose cotransporter 2 inhibitor (SGLT‐2i), glucagon‐like peptide 1 receptor agonist (GLP‐1RA) and dipeptidyl peptidase‐4 inhibitor (DPP‐4i) medicines from 2014 to 2023 in 4 databases.

The completeness of information in ADR reports for each variable across the 4 databases is presented in Figure 2. Information on the primary reporter and outcomes were nearly 100% complete across all 4 databases, with the exception of the Canadian database where outcome was complete in 59% of reports. Sex was reported in >90% of reports across all 4 databases. The FAERS database was the only database that allowed reports from other countries, and so included country source; this variable was complete in 94% (*n =* 253 029) of reports from FAERS. Age was recorded in 3/4 of the reports for the Danish ADR reporting database, UK Yellow Card Scheme and Canada ADR reporting database and (51%) of reports in the FAERS database. Indication for use of the suspected medicine was reported in 59% (*n =* 2532) of reports in the Canadian ADR reporting database and 61% (*n =* 165 351) of reports in the FAERS database. This variable was not available in the publicly available UK Yellow Card Scheme and the Danish ADR reporting database. The least completed variable was for the route of administration, at 44% of reports (*n =* 6013) in the UK Yellow Card Scheme, and for the event date, with 50% (*n =* 135 624) completeness in FAERS (Figure 2).

Where values are not displayed, the data are not reported in the publicly available data for that country.

### 3.3 Trends in the completeness of information in ADR reports over time

**FIGURE 3:**
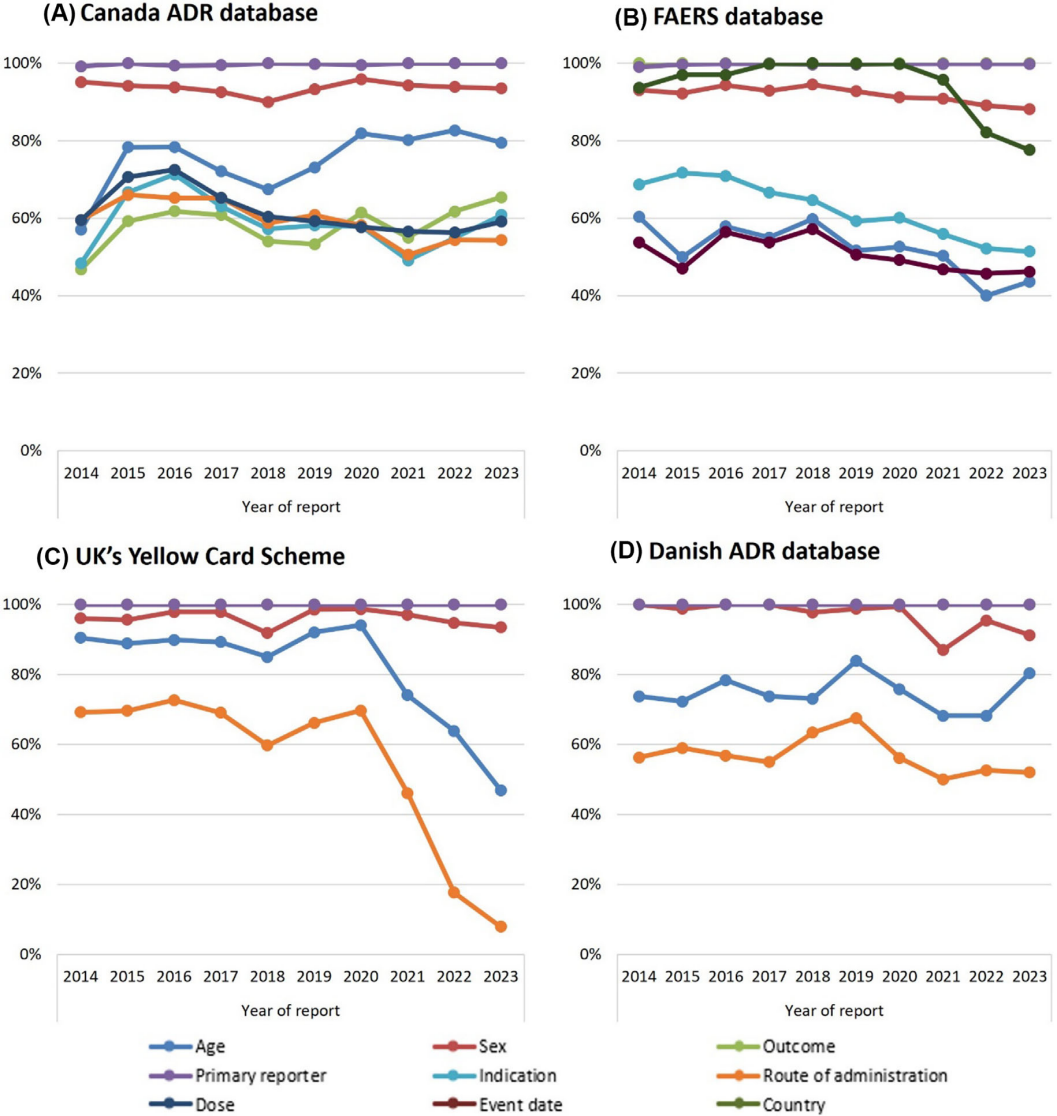
Trends in the information completeness of adverse drug reaction (ADR) reports for sodium–glucose cotransporter 2 inhibitor (SGLT‐2i), glucagon‐like peptide 1 receptor agonist (GLP‐1RA) and dipeptidyl peptidase‐4 inhibitor (DPP‐4i) medicines from 2014 to 2023 in 4 databases.

The completeness of information in ADR reports remained relatively constant from 2014 to 2023 in both the Canada ADR reporting database and Danish ADR reporting database. However, there was a substantial decrease in the completeness of reports in the UK Yellow Card Scheme for 2 variables, age and route of administration, between 2020 to 2023. Additionally, in the FAERS database, there was a slight decrease in completeness for 3 variables, indication between 2016 and 2023, country between 2020 to 2023 and age between 2018 and 2022 (Figure 3).

The average overall completeness score over the study period (2014–2023) was 73% in the Canadian database, 78% in the FAERS database, 83% in the UK database and 85% in the Denmark database.

The analysis of completeness across all fields of studied variables found that all variables were complete in 30% of the reports in both the Canadian and FAERS databases, 40% of reports in the UK database and 51% of reports in the Denmark database (Figure S3).

### 3.4 Completeness of ADR reports according to the seriousness of reports

Overall completeness of ADR reports was higher for serious reports compared to nonserious reports in all databases except for the Canadian ADR reporting database, where overall completeness was higher for nonserious reports.

In the FAERS database, completeness was higher for serious reports compared to nonserious reports for the variables age (68 *vs*. 38%; *P <* .001), indication (69 *vs*. 55%; *P <* .001) and event date (61 *vs*. 42%; *P <* .001).

In the UK database, completeness was higher for serious compared to nonserious reports for age (80 *vs*. 66%; *P <* .001) and route of administration (52 *vs*. 34%; *P <* .001). Similarly, in the Danish ADR reporting database, there was a higher frequency of completeness of age (93 *vs*. 70%; *P <* .001), sex (98 *vs*. 93%; *P <* .001) and route of administration (80 *vs*. 46%; *P <* .001) for serious reports compared to nonserious reports. In contrast, the Canadian ADR reporting database demonstrated less completeness for age (76 *vs*. 82%; *P <* .001), dose (58 *vs*. 76%; *P <* .001), indication (56 *vs*. 76%; *P <* .001) and route of administration (56 *vs*. 68%; *P <* .001) for serious reports comparted to nonserious reports (Table 3).

**TABLE 3:** Completeness of adverse drug reaction (ADR) reports according to the seriousness of the cases using chi‐square test in 4 databases.

Variables	Canada ADR reporting database		FAERS database		UK's yellow card scheme		Danish ADR reporting database	
	Nonserious, *n =* 724	Serious, *n =* 3565	Nonserious, *n =* 150 120	Serious, *n =* 119 643	Nonserious, *n =* 5798	Serious, *n =* 7826	Nonserious, *n =* 1768	Serious, *n =* 635
Age	591 (81.6%)	2705 (75.9%) *	57 605 (38.4%)	80 812 (67.5%) *	383 (66.1%)	6282 (80.3%) *	1243 (70.3%)	588 (92.6%) *
Sex	689 (95.2%)	3326 (93.3%)	137 225 (91.4%)	110 224 (92.1%) *	5548 (95.7%)	7493 (95.7%)	1640 (92.8%)	625 (98.4%) *
Outcome	449 (62.0%)	2094 (58.7%)	–	–	–	–	–	–
Dose	548 (75.7%)	2068 (58.0%) *	–	–	–	–	–	–
Indication	548 (75.7%)	1984 (55.7%) *	82 932 (55.2%)	82 419 (68.9%) *	–	–	–	–
Route of administration	495 (68.4%)	2005 (56.2%) *	–	–	1967 (33.9%)	4046 (51.7%) *	814 (46.0%)	506 (79.7%) *
Primary reporter	721 (99.6%)	3560 (99.9%)	149 861 (99.8%)	119 262 (99.7%) *	–	–	–	–
Event date	–	–	62 478 (41.6%)	73 146 (61.1%) *	–	–	–	–
Country	–	–	144 102 (96.0%)	108 927 (91.0%) *	–	–	–	–

* *P*‐value <.001.

–Variable not reported in that database.

FAERS, Food and Drug Administration Adverse Event Reporting System.

## 4 DISCUSSION

In this study, between 30 and 51% of ADR reports included in the analysis had information recorded in all of the available variables used for undertaking causality assessments between the medicine and the reported ADR. The most frequently omitted information was the route of administration, with completion scores of 44% in the UK Yellow Card Scheme, 55% in the Danish ADR reporting database and 58% in the Canadian ADR reporting database. In the FAERS database event date was the most frequently omitted information (50% complete).

Evaluation of the completeness of ADR reports in the 4 countries showed that reporter type and sex were the most frequently completed information. Conversely, information on route of administration and event date were the most frequently underreported from the available variables in the databases. Consistent with our findings, previous studies conducted in the USA using the FAERS database, in Germany using the EudraVigilance database and in South Africa using the Vigibase reported that event date or time‐to‐onset and route of administration were the most commonly omitted information in ADR reports.^18^, ^40^, ^41^, ^42^, ^43^ This may be due to the retrospective nature of spontaneous reporting, which may make it challenging for reporters to retrieve the date of the event or lack of awareness of the importance of providing this information.^44^, ^45^, ^46^, ^47^, ^48^ A previous study found healthcare professionals reported the process of documenting and reporting ADRs to be time‐consuming in conjunction with their patient care services, which during periods of high activity was often delayed, incomplete or not completed at all.^48^ This finding highlights the need of enhancing awareness among reporters regarding the importance of providing complete information in ADR reports, which is essential for monitoring the safety of medicines.^49^, ^50^

In the vigiGrade completeness tool, the absence of information regarding the time‐to‐onset, which is determined based on medicine start date and event start date, results in a penalty of 50% (Table S2).^11^ In our study, only the FAERS database had the event date available in the publicly accessible database; however, the medicine start date was not available, which precluded the determination of time‐to‐onset. Despite this limitation of the database, our study highlights that since the completeness of the event date was only 50% in the FAERS database, at least 50% of the reports would lack sufficient information to determine time‐to‐onset, which is an essential component for causality assessment. Additionally, incompleteness of each of the variables age, sex, outcome and indication results in a 30% penalty in the vigiGrade completeness score (Table S2).^11^ In our findings, sex and outcome were well reported (between 92 and 96% completeness across databases for sex and 100% completeness across databases for outcome except for the Canadian database). However, completeness of indication, which is available in the Canadian ADR reporting database and FAERS database, was lower, at 59% in the Canadian ADR reporting database and 61% in the FAERS database. Interventions to improve reporting of these variables, including initiatives to raise awareness about the importance of completing this information, are needed to reduce the rate of missing data and ensure sufficient information for causality assessment.

A significantly higher level of report completeness was observed in serious ADR reports compared to nonserious ADR reports in the FAERS database, UK Yellow Card Scheme and Danish ADR reporting database. This is consistent with previous studies conducted in France, Germany and South Africa, which found that serious ADR reports contained more complete information than nonserious reports.^14^, ^18^, ^43^ This may be because consumers and healthcare professionals tend to report serious ADRs promptly after the event.^51^, ^52^, ^53^ Additionally, when a serious ADR is reported to a regulatory authority, the regulatory agency staff will often contact the reporter to request further detailed information about the case to fully understand the severity and determine the causality assessment between the medicine and the reported ADR.^16^ However, in the Canadian ADR reporting database, a significantly higher level of completeness was noted for nonserious ADR reports compared to serious ADR reports. This may because the majority of serious ADR reports in the Canadian database may have been submitted through pharmaceutical manufacturers, where the completeness of reporting was lower than reports directly submitted to regulators, as indicated in previous studies.^17^, ^39^

The completeness of ADR reports from 2014 to 2023 demonstrated a relatively consistent level of information completeness for all variables assessed, except for age and route of administration in the UK Yellow Card Scheme, which exhibited a significant decline in completeness during 2020–2023. We assessed the underlying causes for the substantial decrease in completeness regarding route of administration and age during this period. The absence of an option for specifying the route of administration on the consumer paper‐based ADR reporting form may have been a contributing factor.^54^ However, our analysis revealed that when examining reports submitted by consumers and healthcare professionals independently, we still observed a significant decline in completeness for both age and route of administration. This observation suggests the presence of other factors influencing this issue. However, we were unable to identify the other underlying reasons, and we acknowledge this limitation. We recommend further research to identify the contributing factors.

The overall completeness of all available variables in our study was 30% for both the Canadian and FAERS databases, 40% for the UK database and 51% for the Denmark database. These differences between databases may be attributed to the number and types of variables assessed, as well as the number of ADR reports included from each database. Our findings are comparable to those of a study conducted in Japan, which employed the vigiGrade completeness scoring system and included variables such as time‐to‐onset, indication, outcome, age, sex, dose, primary reporter and report type, reporting that 49.5% of ADRs were well‐documented.^55^ Similarly, a US study that evaluated completeness based on 4 variables including age, sex, event date and at least 1 medical term, found that 41.4% of manufacturer reports were complete, while direct reports by consumers and healthcare professionals had a completeness rate of 86.2%.^41^ In contrast, the completeness observed in our study was higher than that reported in a Portuguese study, which classified reports based on the presence of 5 mandatory variables (patient initials, date of birth, sex, ADR date and drug administration date) and 4 recommended variables (medical history, concomitant medication, clinical evolution and documentation of the indication diagnosis), finding that only 22.4% of reports were well‐documented.^15^ It was also higher than a study conducted in Brazil, which assessed completeness using variables related to patient demographics, drug details (name, dosage, indication, administration route, start/end dates) and ADR information (description, onset/end dates), and found that only 4.4% of reports were fully completed.^17^ Variations in the methods used to assess completeness, the number and types of variables included, the number of ADR reports analysed, and national pharmacovigilance practices probably contribute to the observed differences in the completeness of ADR reports across studies.

While spontaneous ADR reporting plays a vital role in postmarketing drug safety surveillance, we acknowledge that it is subject to limitations and biases. The most significant is under‐reporting, with a systematic review study estimating a median under‐reporting rate of 94%.^44^ Reporting biases can affect the accuracy of the number of ADR reports (numerator), while the absence of reliable drug‐use data (denominator) limits the ability to determine the population at risk, making accurate estimation of ADR incidence difficult.^56^ Variability in under‐reporting across drug classes and countries further complicates comparisons of drug‐safety profiles.^56^ External factors such as media attention, selective reporting of serious ADRs, and consumers and healthcare professionals related factors including time constraints, uncertainty about whether ADR is related to a medicine, and lack of awareness also influence reporting.^56^, ^57^ Therefore, these limitations may affect the apparent frequency and distribution of reported ADRs and should be considered when interpreting the results.

### 4.1 Limitations

This study used elements of the vigiGrade completeness score tool in our completeness assessment; however, we were unable to use the tool in its original validated form for the present study. The vigiGrade completeness tool applies penalties to the completeness score for incomplete information in ADR reports (Table S2). Specifically, the absence of *time to onset* in an ADR report results in a 50% reduction of the completeness score, and *time to onset* was not 1 of the variables available in the publicly accessible datasets that we analysed in this study. The vigiGrade completeness score ranges from 0.07 to 1, with scores greater than 0.8 indicating a well‐documented report^11^ meaning that all reports included in our study would automatically be ineligible to be rated *well documented*. Therefore, a previously validated descriptive completeness assessment approach^39^ was chosen for the present study. We selected this approach because it included variables that are used in the validated vigiGrade completeness score. Another limitation of this study was that the analysed databases did not indicate the reporting methods used, such as paper‐based forms, phone calls or digital tools. Consequently, we were unable to evaluate differences in the completeness of ADR reports based on the reporting method. Identifying differences in report completeness across different methods is essential for determining which approach yields the most complete data, which is important to enhance signal detection. An additional limitation is the potential inclusion of duplicate reports. However, we made efforts to remove duplicates based on the recommendations provided by regulatory authorities and previous studies.^31^, ^32^, ^33^, ^34^, ^35^, ^36^, ^37^, ^38^

## 5 CONCLUSION

The completeness of information in ADR reports varies across different variables. More complete data were reported for primary reporter, outcome (except for the Canadian ADR reporting database), country and sex, as recorded in the 4 databases. In contrast, information was frequently omitted for route of administration, event date, dose and indications as recorded in the 4 databases. To address the issue of missing information, it is important to enhance awareness of providing complete information during ADR reporting to ensure the collection of complete data, thereby facilitating causality assessments and enhancing the pharmacovigilance process.

## AUTHOR CONTRIBUTION

M.G.D. contributed to the conception and design of the study, data analysis, data interpretation, drafting the manuscript and critically revising the manuscript; L.K.E., G.M.K. contributed to the conception and design of the study, data interpretation and critically revising the manuscript; E.A.G. contributed to the design of the study, data interpretation and critically revising the manuscript; R.L. contributed to the conception and design of the study and data interpretation; and E.R. contributed to data interpretation and critically revising the manuscript. All authors read and approved the final manuscript.

## CONFLICT OF INTEREST STATEMENT

The authors declare no conflicts of interest.

## Supporting information


**TABLE S1** Medicines included in the ADR report analysis.
**TABLE S2** Dimensions accounted for in the vigiGrade Completeness score with penalties applied.
**FIGURE S3** Percentage of reports with all fields of analysed variables completed in ADR reports for SGLT‐2i, GLP‐1RA and DPP‐4i medicines from 2014 to 2023 in 4 databases.

## Data Availability

The datasets of the current study are available in the Canada Vigilance Adverse Reaction Online Database, the FAERS of the US, the Yellow Card Scheme of the UK, and the Danish Medicines Agency's Adverse Drug Reaction Database. The data that support the findings of this study are available from the corresponding author upon reasonable request.
