# Supplementary material for: Completeness of spontaneously reported adverse drug reactions in 4 databases
Source: Br J Clin Pharmacol. 2025 Jul 28;91(12):3389–400. doi: 10.1002/bcp.70182 (PMC12648374; doi:10.1002/bcp.70182)
Supplement: Supplementary file 1 — TABLE S1 Medicines included in the ADR report analysis. TABLE S2 Dimensions accounted for in the vigiGrade Completeness score with penalties applied. FIGURE S3 Percentage of reports with all fields of analysed variables completed in ADR reports for SGLT‐2i, GLP‐1RA and DPP‐4i medicines from 2014 to 2023 in 4 databases. [file BCP-91-3389-s001.docx]

**Supplementary material 1**

**Table S1**: Medicines included in the ADR report analysis.

| **SGLT-2is** | **GLP-1RA** | **DPP-4i** | **Medicines in fixed-dose combination form** |
| --- | --- | --- | --- |
| Canagliflozin | Dulaglutide | Sitagliptin | Alogliptin/Metformin |
| Dapagliflozin | Albiglutide | Saxagliptin | Alogliptin/Pioglitazone |
| Empagliflozin | Liraglutide | Linagliptin | Canagliflozin/Teneligliptin |
| Ertugliflozin | Semaglutide | Alogliptin | Dapagliflozin/Metformin/Saxagliptin |
| Ipragliflozin | Exenatide | Vildagliptin | Dapagliflozin/Saxagliptin |
| Luseogliflozin | Lixisenatide | Anagliptin | Empagliflozin/Linagliptin |
| Tofogliflozin |  | Teneligliptin | Empagliflozin/Linagliptin/Metformin |
| Sotagliflozin |  | Evogliptin | Ertugliflozin/Sitagliptin |
|  |  | Gemigliptin | Gemigliptin/Metformin |
|  |  | Omarigliptin | Ipragliflozin/Sitagliptin |
|  |  | Trelagliptin | Linagliptin/Metformin |
|  |  |  | Linagliptin/Pioglitazone |
|  |  |  | Metformin/Saxagliptin |
|  |  |  | Metformin/Sitagliptin |
|  |  |  | Metformin/Vildagliptin |
|  |  |  | Metformin/Teneligliptin |
|  |  |  | Canagliflozin/Metformin |
|  |  |  | Empagliflozin/Metformin |
|  |  |  | Dapagliflozin/Metformin |
|  |  |  | Ertugliflozin/Metformin |

**Supplementary material 2**

**Table S2**: Dimensions accounted for in the vigiGrade Completeness score with penalties applied.

| **Dimension** | **Description** | **Considerations** | **Penalty** |
| --- | --- | --- | --- |
| **Time-to-onset** | Time from treatment start to the suspected ADR. | Imprecise information is penalized if there is ambiguity as to whether the drug preceded the adverse event; with 30% if the uncertainty exceeds 1 month, 10% otherwise. | 50%  30%  10% |
| **Indication** | Indication for treatment with the drug | Penalty imposed if the information is missing or cannot be mapped to standard terminologies such as ICD or MedDRA. | 30% |
| **Outcome** | Outcome of suspected ADR in the patient. | "Unknown" treated as missing. | 30% |
| **Sex** | Patient sex. | "Unknown" treated as missing. | 30% |
| **Age** | Patient's age at onset of the suspected ADR. | Age "unknown" is treated as missing. 10% penalty is imposed if only the age group is specified. | 30%  10% |
| **Dose** | Dose of the drug(s). | Penalty imposed if the total daily dose cannot be calculated from the included fields. | 10% |
| **Country** | Country of origin. | Supportive in causality assessment since medical practice and adverse reaction reporting vary between countries. | 10% |
| **Primary reporter** | Occupation of the person who reported the case (e.g. Physician, Pharmacist). | Supportive in causality assessment since the interpretation of reported information may differ depending on the reporter's qualifications "Unknown" penalized as missing information, whereas "Other" is not penalized. | 10% |
| **Report type** | Type of report (e.g. spontaneous report, report from study, other). | "not available to sender (unknown)" treated as missing. | 10% |
| **Comments** | Free text information. | Uninformative text snippets excluded. | 10% |

**Supplementary material 3**


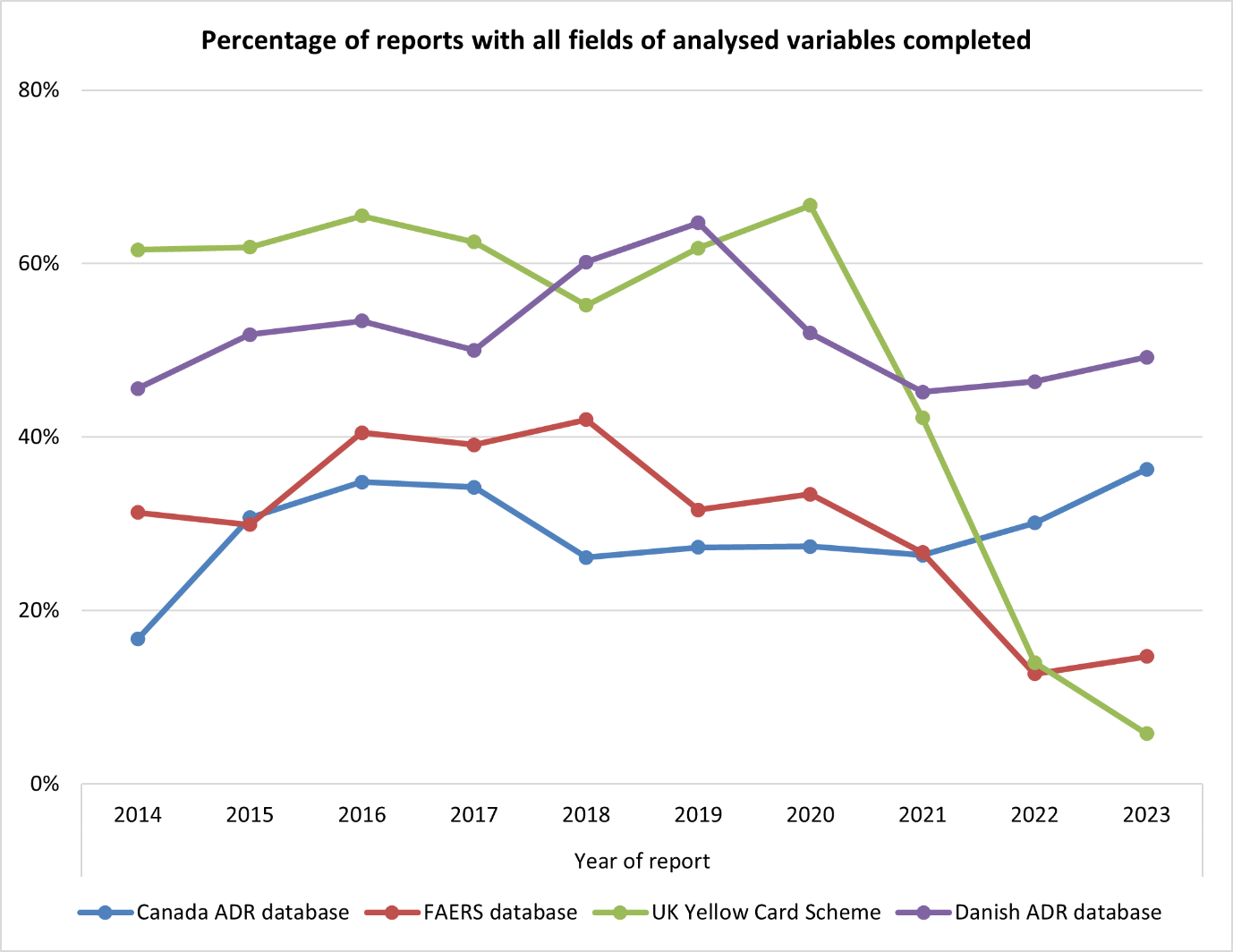
**Figure S3**: Percentage of reports with all fields of analysed variables completed in ADR reports for SGLT-2i, GLP-1RA and DPP-4i medicines from 2014 to 2023 in four database.
